# Triboelectrically-induced non-contact polypropylene/polyvinylidene fluoride sensor with low permittivity supporting layers affecting its interfacial charge dynamics

**DOI:** 10.1038/s41598-026-53473-9

**Published:** 2026-05-19

**Authors:** Petr Slobodian, Pavel Riha, Berenika Hausnerova, Robert Olejnik, Jiri Matyas

**Affiliations:** 1 Centre of Polymer Systems, University Institute, Tomas Bata University, Trida T. Bati 5678, Zlin, 76001 Czech Republic; 2 Department of Physics and Materials Engineering, Tomas Bata University in Zlin, Vavreckova 5669, Zlin, 760 01 Czech Republic; 3 Department of Production Engineering, Faculty of Technology, Tomas Bata University in Zlin, Vavreckova 5669, Zlin, 760 01 Czech Republic

**Keywords:** Triboelectric nanogenerator, Non-contact sensing, Dielectric permittivity, Motion detection, Energy harvesting, Packaging polymers, Energy science and technology, Engineering, Materials science

## Abstract

This work presents a simple and low-cost approach to triboelectric sensing based on commercially available nonwoven polypropylene (PP) membranes paired with electrospun polyvinylidene fluoride (PVDF) to form a triboelectric nanogenerator (TENG), which is further utilized as a sensor operating predominantly in a non-contact regime. The initial contact electrification creates an interfacial charge state that is retained after contact, allowing subsequent separation changes to generate electrical signals via electrostatic induction without requiring continuous contact. The introduction low-permittivity sublayers (polyvinyl chloride (PVC), biaxially oriented polyethylene terephthalate (BOPET), and low-density polyethylene (LDPE)) beneath the tribonegative PVDF membrane significantly enhances the TENG’s mechano-electric performance by modifying the electric field distribution in the multilayer dielectric structure. The PP/PVDF+LDPE layer achieves a peak open-circuit voltage of 689 V and a maximum power density of 5.46 mWcm⁻² in comparison to 376 V and 2.05 mWcm⁻², respectively, for PP/PVDF. This confirms that the dielectric permittivity of the supporting layer is a key parameter controlling the electrical output of the PP/PVDF triboelectric system. The device was validated under various pressure stimuli and vibrations demonstrating its ability to simultaneously sense motion and harvest energy.

**Supplementary Information:**

The online version contains supplementary material available at 10.1038/s41598-026-53473-9.

## Introduction

Polyvinylidene fluoride (PVDF) is one of the most widely used materials in triboelectric nanogenerators (TENGs) due to its efficient electromechanical conversion and strong electronegativity. Electricity generated by mechanical stimuli and harvested by TENGs or used for self-powered sensors^1,2^ is mostly investigated in terms of the used triboelectric material pairs, polarizability, electron affinity, ability to generate sufficient current density and surface charge density^3^. The polarization can be increased by choosing a highly polarized dielectric, such as fluorinated polymers with negatively and positively polarized fluorine and hydrogen atoms, respectively, which enable the formation of dipoles with various configurations^3,4^. An increase in dielectric performance can also be achieved by incorporating nanoparticles into the polymer matrix of the dielectric to moderate the orientation of the dipoles^5^, or by using electrospinning to produce porous dielectric fibrous structures with polymer chains which can modify dipole orientation configurations and increase contact areas^1,3,4,6^. The physical properties of PVDF can be also controlled by rapid or slow cooling during PVDF film fabrication^7^.

A relatively well-explored way to increase PVDF the triboelectric performance is also pairing with PVDF copolymers^8^ or PVDF-based composites^9,10^ and/or incorporation of liquid metals. For example, Galinstan nanodroplets admixed into electrospun PVDF-co-hexafluoropropylene (PVDF-HFP) nanofibers resulted in an open-circuit voltage of 1680 V and a power density reaching 24 W/m² together with other improved properties such as surface potential, capacitance and charge trapping capability^11^. The presence of liquid metal particles contributed to the secondary polarization of PVDF-HFP, which further enhanced its performance. An even higher power density of 56 W/m² was achieved by using a PVDF copolymer with poly(diethylene glycol methyl ether methacrylate) in combination with tribo-negative polydimethylsiloxane as the second active TENG layer^12^. The synthesis of the PVDF copolymer resulted in to the transformation of the tribo-negative original PVDF into a tribo-positive PVDF copolymer.

It should also be emphasized that PVDF has inherent piezoelectric properties^13,14^. In combination with other materials, the triboelectric properties of PVDF can be studied in the course of piezo-tribo hybrid nanogenerators (PTENG)^14–17^. Other materials used as the second part of the triboelectric pair include poly(tetrafluoro ethylene) PTFE^14^, polydimethylsiloxane (PDMS)^16^ and biaxially oriented poly(ethylene therephthalate) (BOPET)^17^. The piezo-tribo hybrid nanogenerator made of a PVDF film with incorporated ZnO nanoparticles coupled with PTFE achieved a maximum output power 2.5 times higher than that of pure PVDF, reaching a value of 24.5 µW/cm²^14^. ZnO nanoparticles have also been applied in the PTENG based on PVDF/ZnO-PDMS/rGO nanocomposites^15^.

However, in this work, commercially available polymers, rather than using complex synthesized materials, are investigated. The need to address the growing problem of plastic packaging waste opens a relatively unexplored way for low-cost TENG design that utilizes available, durable, and cost-effective commercial plastics. Recent studies show that a wide range of post-consumer plastics can be reused for triboelectric power generation, providing a sustainable alternative to conventional fluoropolymers^17–24^. Unsorted plastic packaging waste consisting of polyethylene (PE), polypropylene (PP), polystyrene (PS) and PET has been shown to generate substantial triboelectric, piezoelectric and pyroelectric outputs, with heterogeneous blends often outperforming single polymers due to interfacial interactions between immiscible phases^19^.

Immiscible blends of post-consumer thermoplastics have been shown to enhance electricity generation in PVDF-based harvesters, where phase separation and interfacial polarization significantly increase the electromechanical response^20^. Other approaches use recycled polymer laminates or waste-derived PET- and PS-based structures to construct tribo- or piezo-tribo hybrid nanogenerators with performance comparable to virgin materials^21^. Single-polymer waste films, such as biaxially oriented polypropylene (BOPP) from packaging sources have also been identified as efficient mechano-electric transducers suitable for energy harvesting^22^.

Moreover, recycled waste streams of PET, PP, polyvinyl chloride (PVC) and high-density PE (HDPE) have been shown to form triboelectric pairs, the polarity arrangement and dielectric contrast of which determine the achievable voltage output in sustainable TENG assemblies^23^. In particular, we previously reported that biaxially-oriented PET films used worldwide as food packaging materials combine unique dual electrification properties that enable efficient conversion of mechanical energy to electricity in a coupled piezo-triboelectric nanogenerator^17^. Overall, these studies confirm that post-consumer plastics represent a technically viable and widely available resource for the construction of TENG devices, supporting their use in low-cost energy harvesting and self-powered sensing applications.

In addition to selection of tribopairs, the dielectric environment of the triboelectric layers has been identified as a key parameter affecting the TENG output. Both theoretical modelling and experimental reports suggest that introduction of a low-permittivity support layer under the tribonegative dielectric limits dielectric screening of the surface charge, and thus increases the electric field intensity reaching the electrode, leading to a higher open-circuit voltage^25–28^. This approach is particularly attractive for commodity packaging polymer films (later available as waste streams), which serve simultaneously as a mechanical support and dielectric enhancement layers. Min et al.^25^ showed that replacing the PET supporting layer (*ε* ≈ 3.2) with lower-permittivity PTFE (*ε* ≈ 2.1) increased the open-circuit voltage by approximately 1.3 in a contact-separation TENG, confirming the theoretical prediction from the equivalent permittivity model. Importantly, the use of high-permittivity layers would have the opposite effect, as higher permittivity of the layer increases dielectric screening and reduces the electric field at the electrode^29^.

The electrical response of triboelectric systems is governed by the electrostatic field associated with the interfacial charge. When an external circuit is present, this field induces charge a redistribution between the electrodes. In predominantly non-contact sensing configurations, the retention of interfacial charge after the initial electrification step enables signal generation via electrostatic induction without the need for continuous mechanical contact^30–34^.

In this study, we develop a triboelectrically-induced non-contact sensor for motion monitoring that can simultaneously harvest electric energy from motion using paired triboelectric polymers. Unusually for a PVDF-based TENG, we paired an electrospun tribonegative PVDF membrane with a spun-bond PP membrane as the tribopositive layer - both of which are commercially available and used for FFP2 respiratory masks production and agricultural foils, respectively. The output of this PP/PVDF pair is further enhanced by introducing low-permittivity supporting sublayers (PVC, BOPET and LDPE) derived from commodity packaging plastics beneath the PVDF membrane. The selection of these sublayers represents both a suitable permittivity choice and a practically relevant solution within a waste-plastic-based TENG future approach.

## Experimental

### Materials

The dielectric PVDF polymer was purchased from Arkema (Kynar Flex^®^ 2801, Zhejiang, China). PVDF nonwoven fiber networks were prepared by electrospinning a 20 wt% solution of PVDF in N, N-dimethylformamide (2 g PVDF in 8 g DMF) using a multi‑needle setup operated at 65 kV, a needle–collector distance of 20 cm and a solution feed rate of 2.3 cm³h⁻¹. The PVDF nanofibers were collected during processing on spun-bond nonwoven dielectric polypropylene (PP). Detailed processing conditions and further details regarding PVDF fibers and PVDF non-woven networks can be found in our previous papers^20^. Spun-bond nonwoven PP membranes with an area weight of 30 g/m^2^ and thickness 0.3 mm were purchased from Pegatex S, PFNon-wovens s.r.o., Znojmo, Czech Republic. The PP membranes were used not only as a collecting substrate during the electrospinning process of the PVDF networks, but also as a triboelectric layer itself. The measured thickness and calculated porosity of the PP membrane were 300 ± 30 μm and 0.82 ± 0.02, respectively. The porosity of the PVDF membrane was 0.88 ± 0.01. The apparent densities of PVDF and PP membranes were 0.32 ± 0.04 g/cm^3^ and 0.12 ± 0.01 g/cm^3^, respectively. The mass density of PVDF was (according to the supplier) 1.78 g/cm^3^, and the PP mass density of compression molded film from PP membrane at 170 °C was 0.92 g/cm^3^.

**Fig. 1:**
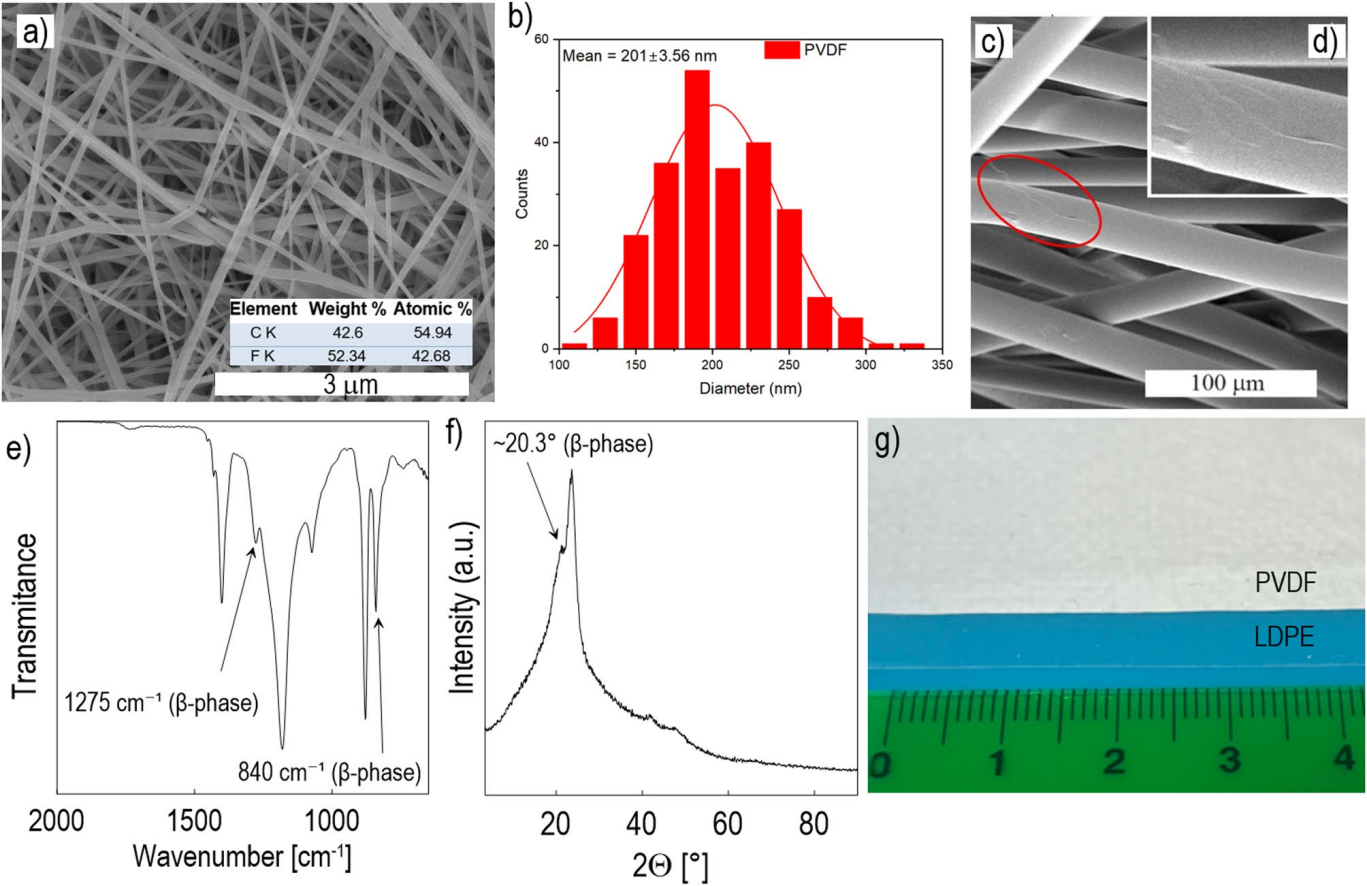
Structural and morphological characterization of the electrospun PVDF membrane: (**a**) SEM of porous nanofibrous network with EDX confirming presence of C and F, (**b**) fiber diameter distribution, (**c**) SEM image of spun-bond PP membrane with (**d**) detail showing PVDF nanofiber adhered to PP fiber surface, (**e**) FTIR spectrum indicating characteristic peaks of PVDF β-phase, (**f**) XRD pattern confirming PVDF β-phase, (**g**) photograph of PVDF membrane electrostatically adhered to LDPE sublayer.

The morphology and structure of the electrospun PVDF membrane are shown in Fig. 1. Scanning electron microscope (SEM, NOVA NanoSEM 450 from FEI Co., Hillsboro, OR, USA) operating at an accelerating voltage of 10 kV, was used to analyze the nanofibrous network with randomly oriented fibers and high porosity, Fig. 1a. The chemical composition of the membrane was confirmed by Energy Dispersive X-ray Spectroscopy (EDX) (inset in Fig. 1a), which shows the expected presence of carbon and fluorine consistent with a PVDF structure. The corresponding fiber diameter distribution (Fig. 1b) shows an average diameter of approximately (201 ± 3.6) nm. An SEM image of the spun-bond PP membrane used as a substrate for electrospinning is depicted in Fig. 1c with residual PVDF nanofibers remaining partially attached to the PP fibers after membrane removal (in detail in Fig. 1d). Fourier transform infrared spectra (FTIR, Thermo Scientific Nicolet iS50), Fig. 1e, exhibit characteristic absorption bands at 840 cm⁻¹ and 1275 cm⁻¹, which are attributed to the electroactive *β*-phase of PVDF. This claim is further supported by X-ray diffraction (XRD) analysis carried out using a MiniFlex™ diffractometer (Malvern Panalytical, Malvern, UK) with CoKβ radiation at 40 kV and 15 mA. The sample was scanned over the 2θ range of 10–90°, and the collected data, originally obtained using a cobalt source, were subsequently converted to a copper source using PowDLL software. The XRD pattern (Fig. 1f) shows a broad diffraction maximum in the range of ~ 20–22° (2θ). The position at ~ 20.3° corresponds to the (110)/(200) reflection of the β-phase of PVDF. Finally, Fig. 1g presents a photograph of the PVDF membrane electrostatically adhered to the LDPE film (low-permittivity film).

Three types of dielectric polymeric films made of low-density polyethylene (LDPE), biaxially oriented polyethylene terephthalate (BOPET) and polyvinyl chloride (PVC), used as food packaging plastics, were purchased from Fatra a.s., Napajedla, Czech Republic. Films with a thickness of 150 μm, measured with a micrometer, were used as low-permittivity layers underlying the PVDF networks in the dielectric-to-dielectric triboelectric nanogenerator (TENG).

Dielectric analysis (DEA) was performed using a broadband dielectric/impedance analyzer (Novocontrol Technologies GmbH & Co. KG, Montabaur, Germany). The samples were placed between two gold-plated electrodes with a diameter of 20 mm, and the relative permittivity and dielectric loss were measured in the frequency spectrum from 1 Hz to 1 MHz at room temperature. To determine the standard surface roughness parameters Sa, arithmetic mean height; Sq, root mean square height; and Sz, maximum height according to the ISO 21920-2 standard (2023) a 3D scanner (Zygo NewView 8000, AMETEK, Inc., Middlefield, Connecticut, USA) with Coherence Scanning Interferometry (CSI) technology was used, which combines VSI - Vertical Scanning Interferometry and PSI - Phase Shifting Interferometry.

### Contact electrification measurements

Two types of mechanical excitations were employed. Manual excitation (hand tapping) was used to generate high-impact conditions, reaching a maximum contact pressure of approximately 270 kPa and controlled cyclic measurements, which were performed using a solenoid-driven actuator operating at a frequency of 10 Hz, providing reproducible contact–separation cycles with an applied pressure of 14.8 kPa and an active area of 5 cm². The solenoid actuation ensures stable and repeatable loading conditions by eliminating operator-introduced variability.

**Fig. 2:**
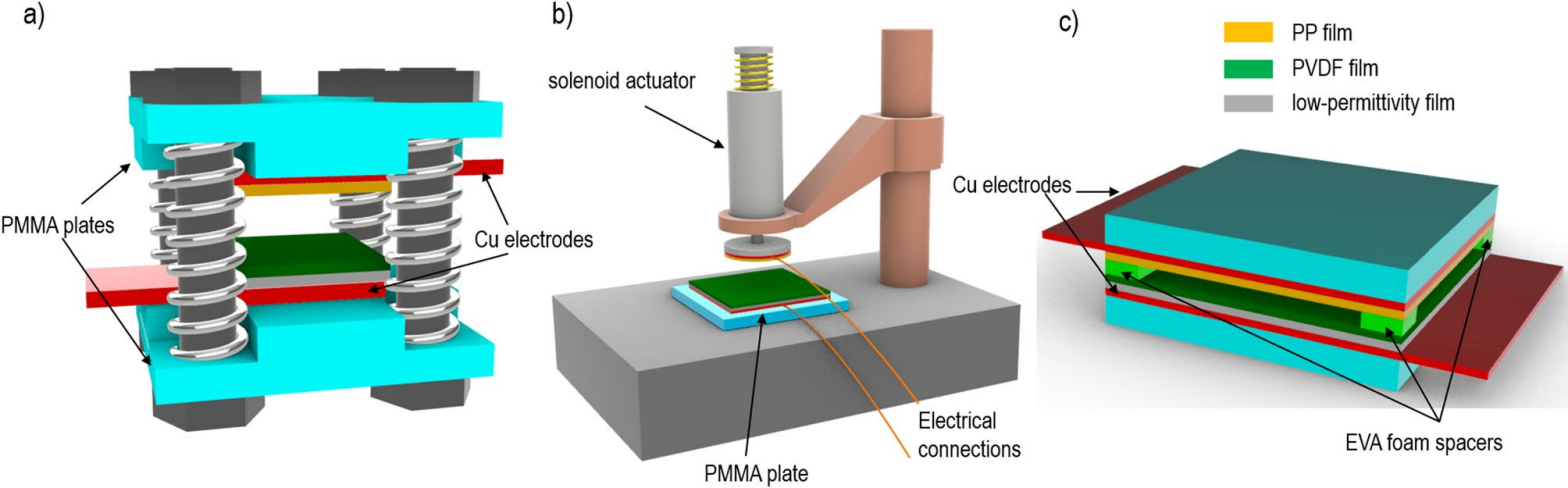
Schematic of TENG and measurement configurations: (**a**) vertical contact–separation setup with spring-guided PMMA plates, (**b**) solenoid-driven contact–separation setup (**c**) non-contact sensing configuration.

The schematic of the manual TENG setup is shown in Fig. 2a. The device operates in vertical contact–separation mode to evaluate electrification of the PP/PVDF triboelectric pair. It consists of two PMMA plates (60 × 60 mm, thickness 3 mm) guided by four metal rods with springs that allow controlled movement and contact between the plates. The initial separation distance between the plates was 6 mm. Both plates were equipped with Cu electrodes made of conductive adhesive Cu tape. The PP spun-bond membrane was attached to one electrode, while the electrospun PVDF membrane was fixed to the opposite electrode. The effective contact area of the TENG, defined by the overlap of the polymer layers, was 6 × 3 cm (18 cm²).

The solenoid-driven TENG setup is shown in Fig. 2b. In this configuration, the bottom electrode was mounted on a fixed PMMA substrate, while the top electrode was attached to a steel actuator head driven by a computer-controlled solenoid, ensuring reproducible and operator-independent contact–separation cycles. The PP membrane was fixed to the moving top electrode, while the PVDF membrane was attached to the bottom electrode. The effective contact area in this arrangement was 5 cm².

Both configurations operate in the same vertical contact–separation mode and were used for load-resistance characterization. In addition, the solenoid-driven setup was used to measure long-term stability (up to 50,000 cycles) and charge density determination due to its excellent reproducibility. Low-permittivity sublayers (LDPE, BOPET or PVC films) were introduced under the PVDF membrane to investigate their effect on the triboelectric output.

The generated triboelectric output voltage was measured with an oscilloscope (Infinivision 1000 x-series, 4ch, 100 MHz, DSOX1204A, Keysight, Santa Rosa, CA, USA) in combination with a high-impedance differential probe to minimize loading effects of the measurement system. In all experiments, the anode was connected to the tribonegative PVDF layer. The load-dependent electrical response was evaluated by connecting the TENG to the external resistive loads in the range of 2.2 kΩ–60 MΩ. The voltage across each load was measured with an oscilloscope connected in parallel to the resistor. The current density was calculated from Ohm’s law as *J* = *V*/(*R*·A), where *V* is the measured voltage, *R* is the external resistance and *A* is the active device area. The power density was determined as *P* = *V*²/(*R*·*A*). Finally, the generated charge density *Q* was measured using an electroscope-charge GRG-BTA sensor connected to a LabQuest interface system (Vernier, Edufor s.r.o., Prague, Czech Republic). The TENG was equipped with a strain gauge L6D-C3-40 kg (Zemic Europe B.V., Etten-Leur, The Netherlands) to measure the force applied in contact with the plates. An analog converter TZA11410 (VTS Zlin s.r.o., Zlin, Czech Republic) was used to power it with a ± 20 mA output and 24 V DC supply.

### Non-contact measurements

The non-contact triboelectric sensor and the associated energy harvesting configuration are schematically shown in Fig. 2c. The device consists of two PMMA plates (60 × 60 mm, thickness 3 mm) equipped with Cu electrodes and triboelectric layers, where the PP spun-bond membrane is attached to the top electrode and the electrospun PVDF membrane to the bottom electrode, with a low-permittivity sublayer placed under the PVDF. The distance between the plates is defined by spacers made of ethylene vinyl acetate (EVA) copolymer foam. Four spacers (3 mm thick, (5 × 5) mm) were placed at the corners of the plates, maintaining a defined distance and acting as compliant spring elements that control the distance between the triboelectric layers during compression. The operating principle of the sensor is based on electrostatic induction driven by pre-existing triboelectric charges at the PP/PVDF interface.

The sensor response was evaluated under various mechanical excitations, including controlled periodic loading using a solenoid actuator (10 Hz), impact from free-fall ball, vibration stimuli generated by a rotary vacuum pump and during sieve analysis, controlled excitation using a miniature eccentric rotating mass vibration motor placed on the top electrode, ultrasonic excitation produced by a sonication horn and cyclic human stepping. These experiments demonstrate the capability of the sensor to detect a wide range of pressure and vibrational inputs.

In addition to sensing, the generated electrical signal was used to obtain energy. The device was actuated using a solenoid with a frequency of 10 Hz and an applied pressure of approximately 3 kPa. The output signal was rectified by a Graetz bridge consisting of four Schottky diodes (forward voltage 1.5 V) and used to charge external film capacitors with capacitances in the range of 10–128 nF. The charging behavior was evaluated as a function of the number of excitation cycles. After charging, the stored energy was released through a 10 MΩ resistive load to analyze the discharge characteristics and estimate the energy storage capability of the system. As a proof-of-concept of direct energy utilization, the rectified signal was sufficient to intermittently power 18 LEDs, producing visible blinking under 10 Hz solenoid excitation (Supplementary Video V1).

## Results

### Electrical response and effect of low-permittivity sublayers

**Fig. 3:**
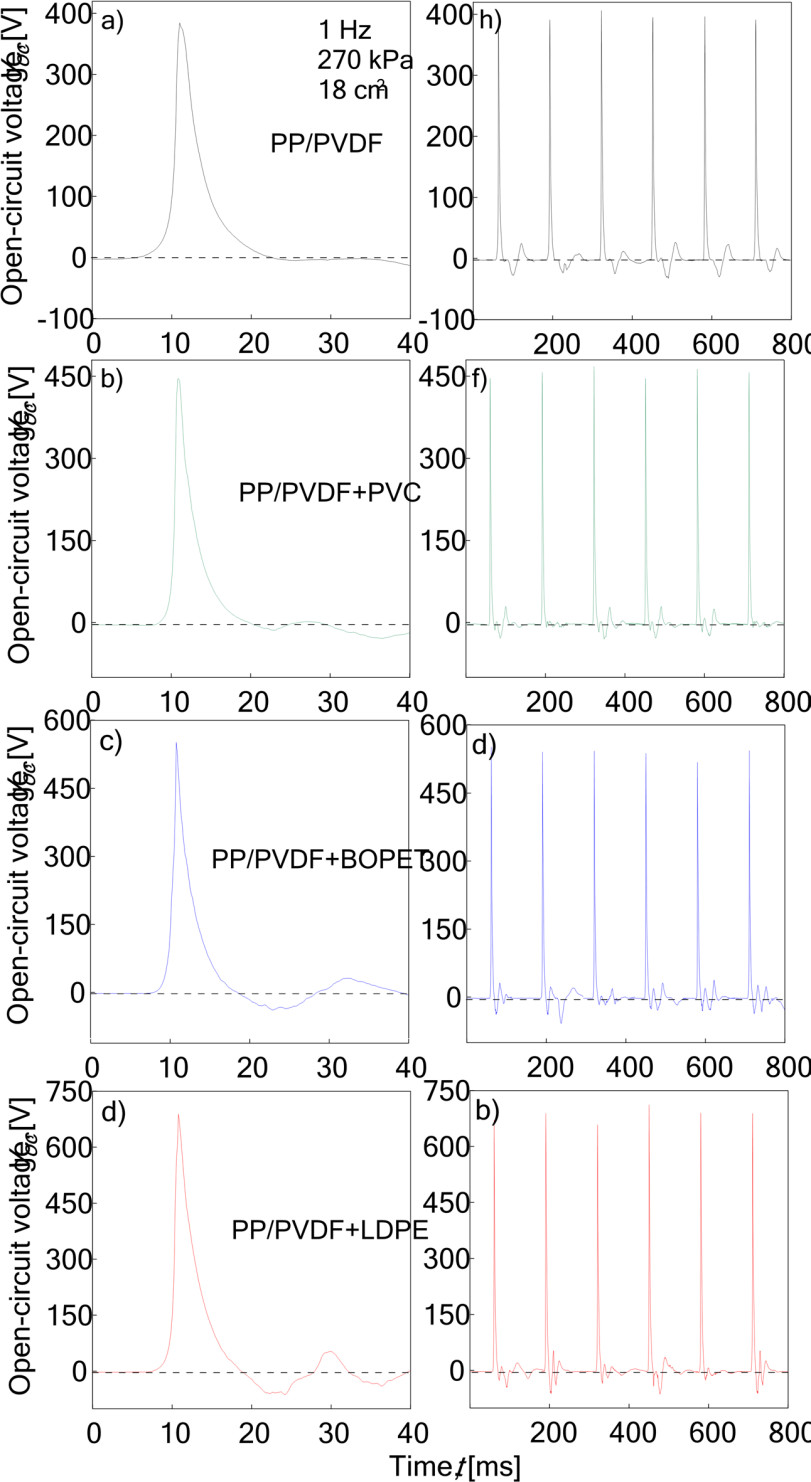
Open-circuit voltage (*V*oc) generated by a single contact (left) and voltage response during six consecutive contact–separation cycles (right) of PP/PVDF-based devices with LDPE, BOPET, and PVC supporting layers (manual set-up, 1 Hz, 270 kPa, 18 cm^2^).

The electrical performance of the PP/PVDF-based triboelectric system was first evaluated in the contact–separation mode using the manual setup under high-pressure excitation. The effect of low-permittivity supporting sublayers (PVC, BOPET, and LDPE) on the electrical response was systematically investigated and compared to the reference PP/PVDF configuration. Representative voltage outputs for single contact events and consecutive contact–separation cycles are shown in Fig. 3 (TENG photograph can be seen in Supplementary Material Fig. S1).

A single contact generates a characteristic open-circuit voltage (*V*oc), followed by a series of six consecutive contact–separation cycles, indicating stable and repeatable signal generations. The peak open-circuit voltage strongly depends on the type of sublayer. The reference PP/PVDF device reaches a maximum *V*oc of approximately 376 V, while the introduction of low-permittivity sublayers leads to a systematic increase to ~ 443 V for PVC, ~ 540 V for BOPET, and even up to ~ 689 V for LDPE. This trend clearly shows that decreasing the permittivity of the supporting layer enhances the induced voltage, which is consistent with the proposed mechanism.

**Fig. 4:**
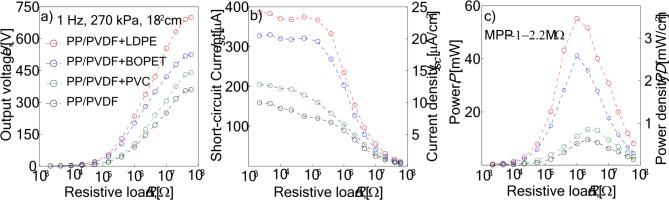
Restive load dependent characterization of the PP/PVDF-based TENGs with low-permittivity LDPE, BOPET and PVC sublayers: (**a**) output voltage, (**b**) short-circuit current density, and (**c**) output power density under mechanical excitation (manual set-up, 1 Hz, 270 kPa, 18 cm^2^).

The electrical performance of the PP/PVDF-based TENG was evaluated through a load-resistance sweep as shown in Fig. 4. With increasing external resistance, the output voltage increases while the current decreases, which corresponds to the typical behavior of triboelectric nanogenerators. The output power exhibits well-defined maximum power peaks (MPP) ranging from 1 to 2.2 MΩ. Under these conditions, the PP/PVDF provides a peak power density of ~ 0.59 mWcm⁻², which increases significantly with the introduction of low-permittivity sublayers to ~ 0.84 mWcm⁻² (PVC), ~ 2.58 mWcm⁻² (BOPET), and up to ~ 3.45 mWcm⁻² for the LDPE. These results clearly demonstrate that the introduction of low-permittivity supporting layers significantly increases the electrical output of the device.

**Fig. 5:**
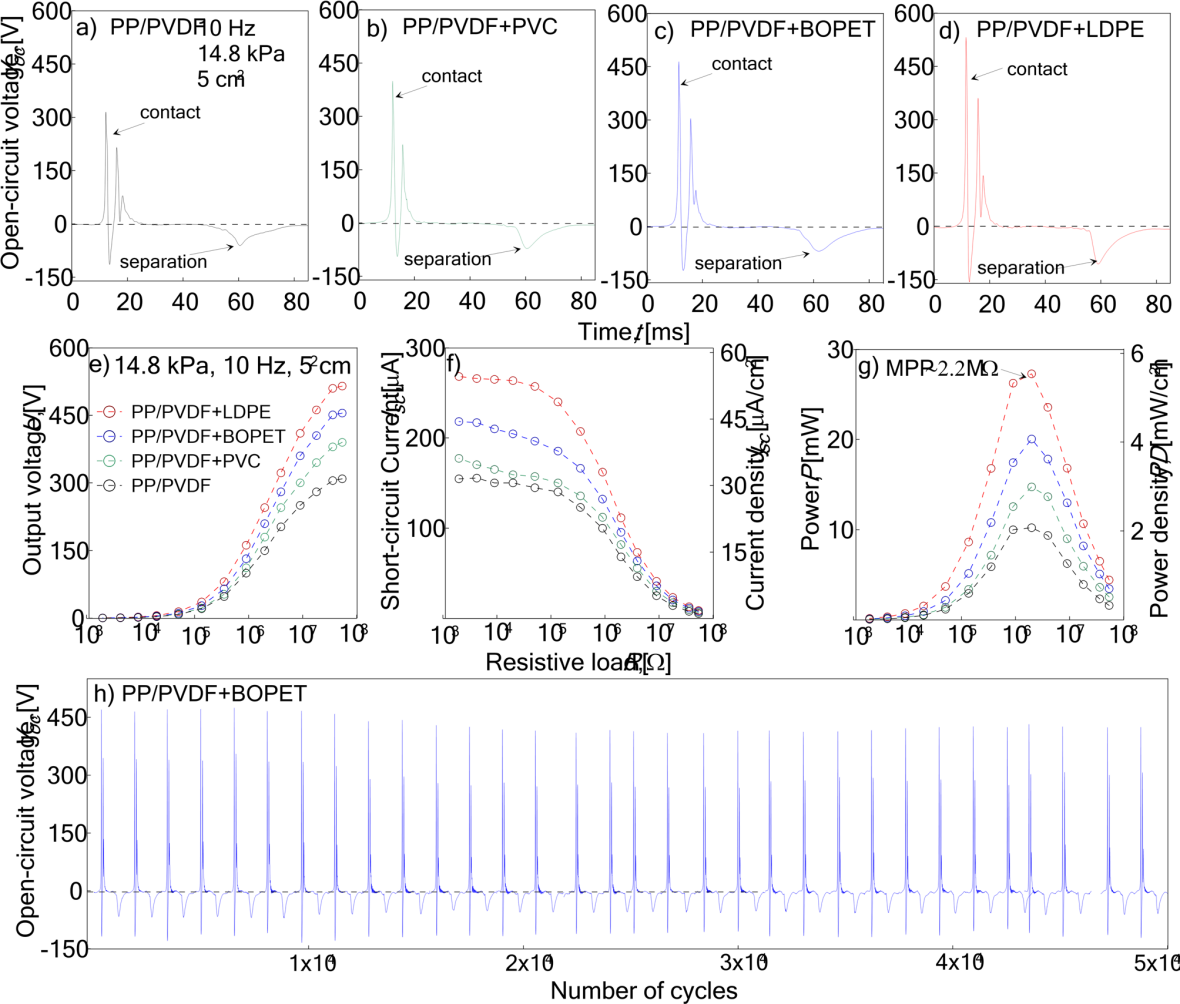
Electrical performance of PP/PVDF-based TENG with low-permittivity LDPE, BOPET and PVC sublayers under contact–separation: open-circuit voltages (**a–d**), resistive load dependent output voltage (**e**), short-circuit current and current density (**f**), power and power density (**g**), and time-resolved voltage output for the PP/PVDF+BOPET device demonstrating stable operation over 50,000 cycles (**h**); (solenoid-driven excitation, 10 Hz, 14.8 kPa, 5 cm^2^).

Figure 5 shows the open-circuit voltage response for individual contact–separation events under solenoid-driven excitation (10 Hz, 14.8 kPa, 5 cm²) for the PP/PVDF TENG and devices with LDPE, BOPET, and PVC sublayers. In all cases, a sharp positive peak during contact is followed by a broader negative signal associated with separation. The peak open-circuit voltage *V*_OC_ increases with decreasing permittivity of the supporting layer and reaches approximately 314 V (PP/PVDF), 392 V (PVC), 464 V (BOPET) and up to 531 V for the LDPE-based device.

The load-dependent electrical characteristics are presented in Fig. 5d–f. The output voltage increases with the external resistance (Fig. 5d), while the current density decreases accordingly (Fig. 5e), reflecting the typical behavior of triboelectric generators. The corresponding power density (Fig. 5f) shows a clear maximum at the optimal load resistance of approximately 2.2 MΩ. Under these conditions, the peak power density increases from ~ 2.05 mWcm⁻² (PP/PVDF) to ~ 2.95 mWcm⁻² (PVC), ~ 4.01 mWcm⁻² (BOPET), and up to ~ 5.46 mWcm⁻² for the LDPE-based device. The corresponding current densities are approximately 31, 34, 43, and 53 µAcm⁻², respectively.

**Fig. 6:**
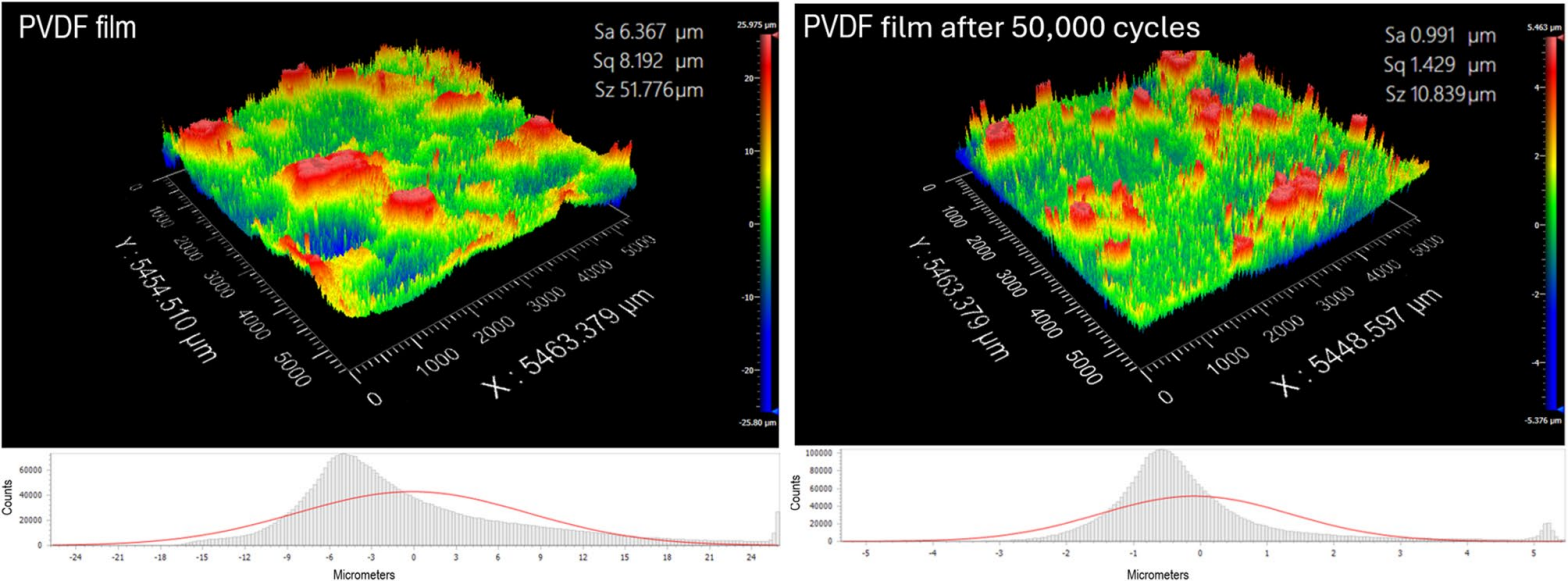
Surface topography of the electrospun PVDF membrane before and after 50,000 contact–separation cycles (solenoid-driven excitation, 10 Hz, 14.8 kPa, 5 cm^2^).

Figure 5h shows the long-term stability of the device for the PP/PVDF+BOPET over 50,000 consecutive contact–separation cycles. The periodic voltage output confirms stable and repeatable signal generation without noticeable degradation or delamination, highlighting durability of the device under dynamic excitation. To further confirm the mechanical durability of the electrospun PVDF membrane, its surface topography (Fig. 6) was examined before and after 50,000 contact–separation cycles. Although partial smoothing of the surface features was observed after cycling, no loss of macroscopic membrane integrity was detected, which is consistent with the stable electrical output.

**Fig. 7:**
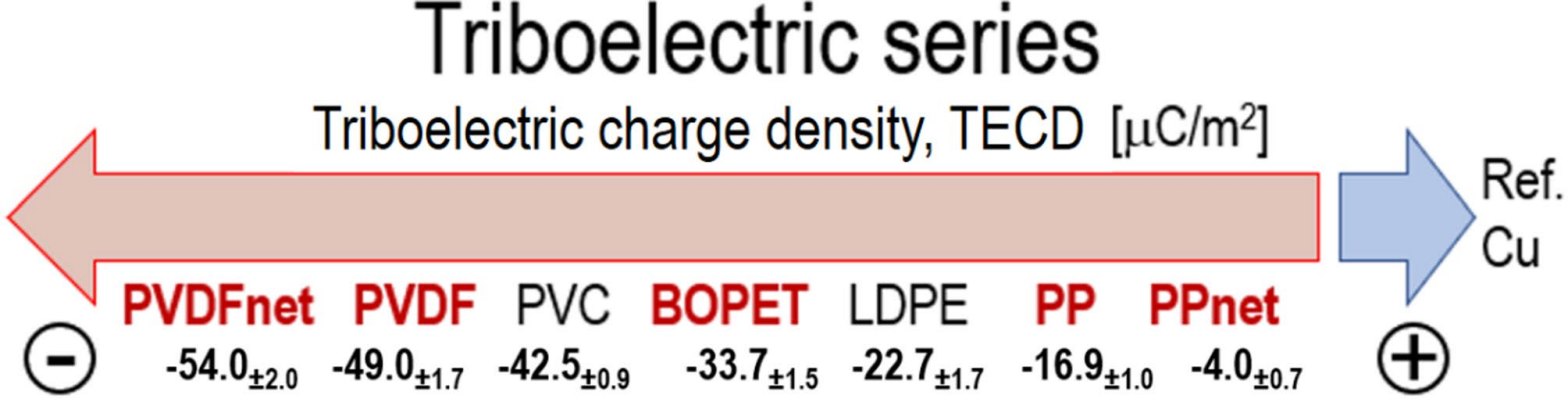
Triboelectric series of PVDF, PP, and low-permitivity sublayers (LDPE, BOPET, PVC) obtained from triboelectric charge density measurements relative to copper reference.

To relate the measured electrical outputs to the triboelectric properties of the tested polymers, a reference triboelectric series was created by repeatedly contacting each polymer with a copper (Cu) electrode. Copper served as a stable tribopositive reference material. All of the investigated packaging polymers exhibited negative charge transfer with respect to Cu, which allowed them to be ranked relative to each other according to the magnitude of the generated charge density. The resulting sequence (Fig. 7) provides a comparative scale of tribonegativity with respect to Cu.

### Dielectric properties and mechanism of sublayer enhancement

**Fig. 8:**
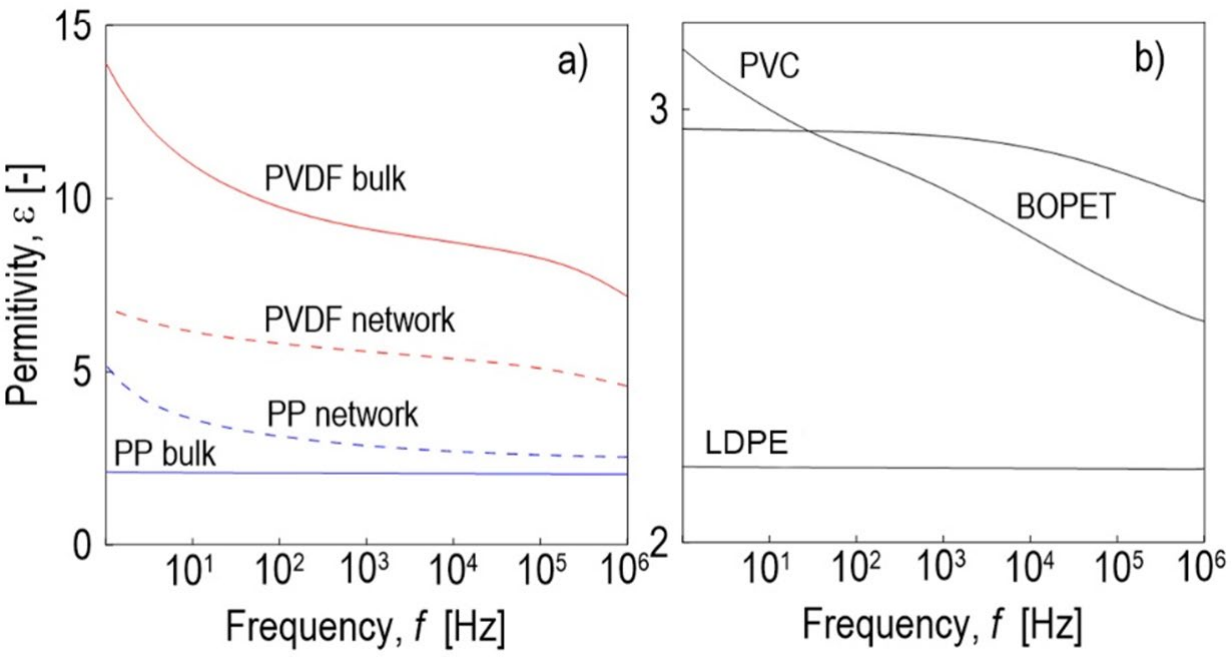
Dielectric relative permittivity of (**a**) PP and PVDF triboelectric pair and (**b**) PVC, BOPET and LDPE sublayers as a function of frequency at room temperature.

Dielectric analysis was used to determine the relative permittivity of the PVDF and PP membranes and the PVC, BOPET, and LDPE sublayers. The frequency-dependent permittivity is shown in Fig. 8. At 1 Hz, the relative permittivity values were 6.85 for the PVDF membrane, 5.08 for the PP membrane and 3.14, 2.95, and 2.16 for PVC, BOPET and LDPE films, respectively. These results confirm that the introduced sublayers possess significantly lower permittivities than the PVDF membrane.

To quantify the effect of the supporting layer, the effective permittivity of the tribonegative PVDF-based bilayer was estimated using an equivalent capacitor model:

1 $$\:{\epsilon\:}_{r,eg}=\frac{{\epsilon\:}_{r,PVDF}{\epsilon\:}_{r,SUB}\left({d}_{PVDF}+{d}_{SUB}\right)}{{d}_{PVDF}{\epsilon\:}_{r,SUB}+{d}_{SUB}{\epsilon\:}_{r,PVDF}}$$

where *ε*_r, PVDF_ and *ε*_r, SUB_ are the relative permittivities of the PVDF membrane and sublayers and *d*_PVDF_ and *d*_SUB_ represent their thicknesses. The calculated equivalent permittivity decreases from 6.85 (PVDF alone) to 5.20 (PVC), 5.05 (BOPET), and 4.33 (LDPE) reflecting the increasing dielectric contrast introduced by the sublayers.

**Fig. 9:**
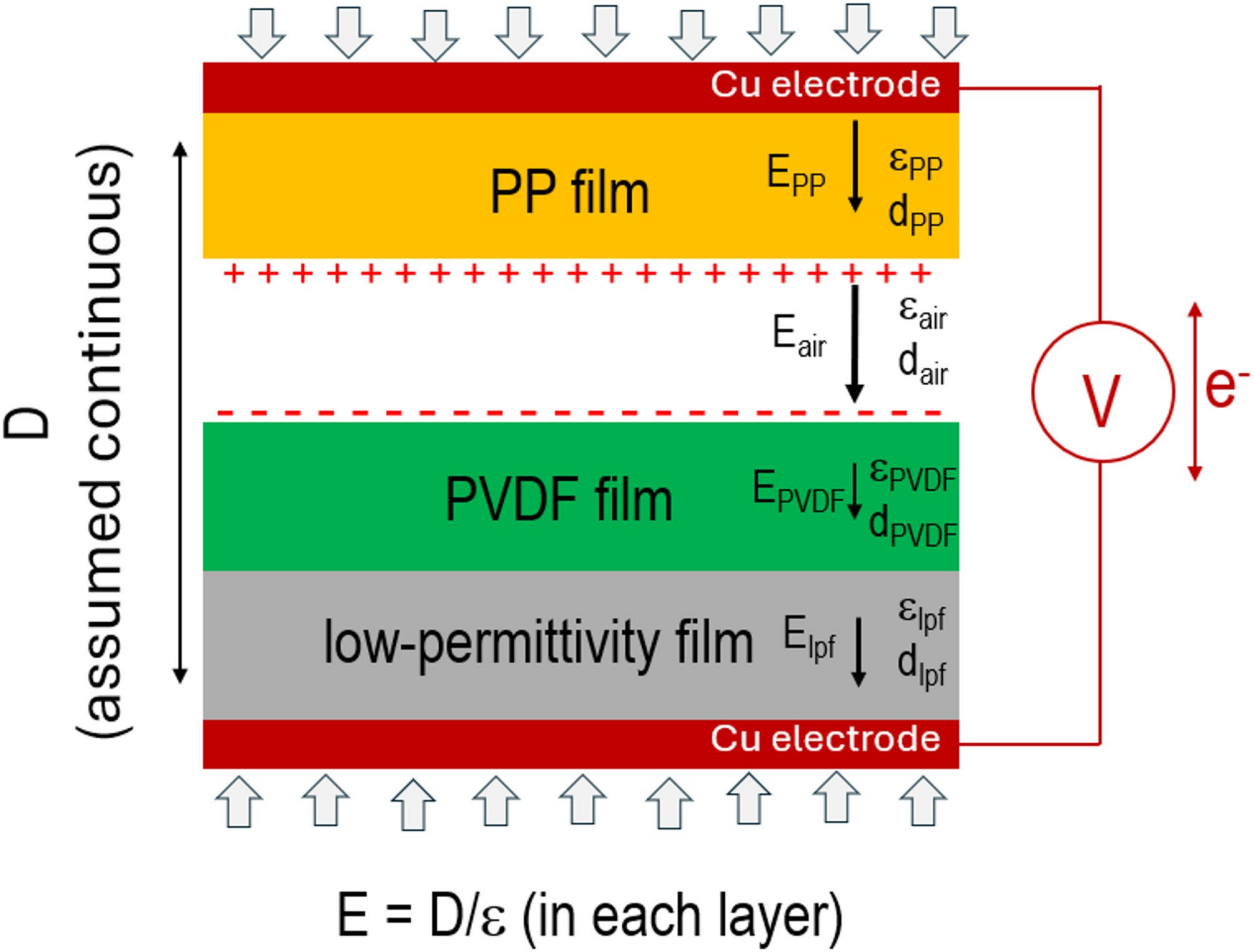
Schematic representation of the multilayer dielectric model illustrating triboelectric charge generation at the PP/PVDF interface and the resulting electric field distribution across the dielectric layers (simplified model assuming continuous displacement field).

The experimentally observed increase in output voltage with decreasing sublayer permittivity is consistent with a decrease in the equivalent permittivity. A lower equivalent permittivity reduces the dielectric screening of the interfacial charge and enhances the electric field at the electrode. This behavior can be interpreted using a simplified multilayer dielectric model (Fig. 9), in which the displacement field is assumed to be approximately continuous across the dielectric layers, while the electric field scales inversely with permittivity (*E* = *D*/*ε*). As a result, low-permittivity sublayers support higher electric fields, leading to stronger electrostatic induction and increased voltage output, in agreement with the observed ordering LDPE > BOPET > PVC.

### Non-contact sensing and displacement-dependent response

When the triboelectric layers are separated by an air gap, the pre-existing triboelectric charges at the PP/PVDF interface generate an electric field across the gap. Changes in the separation distance during relative motion modulate this field and induce voltages on the electrodes via electrostatic induction^35^. To quantify this effect under well-defined conditions, measurements were performed using the contact–separation setup (Fig. 2a), in which the separation distance was externally controlled by calibrated steel spacers placed outside the active area of the triboelectric layers. This configuration allows for precise adjustment of the air gap without changing the mechanical compliance of the system and distinguishes this controlled experiment from the non-contact sensing configuration shown in Fig. 2c, where compliant EVA spacers define the gap during operation.

**Fig. 10:**
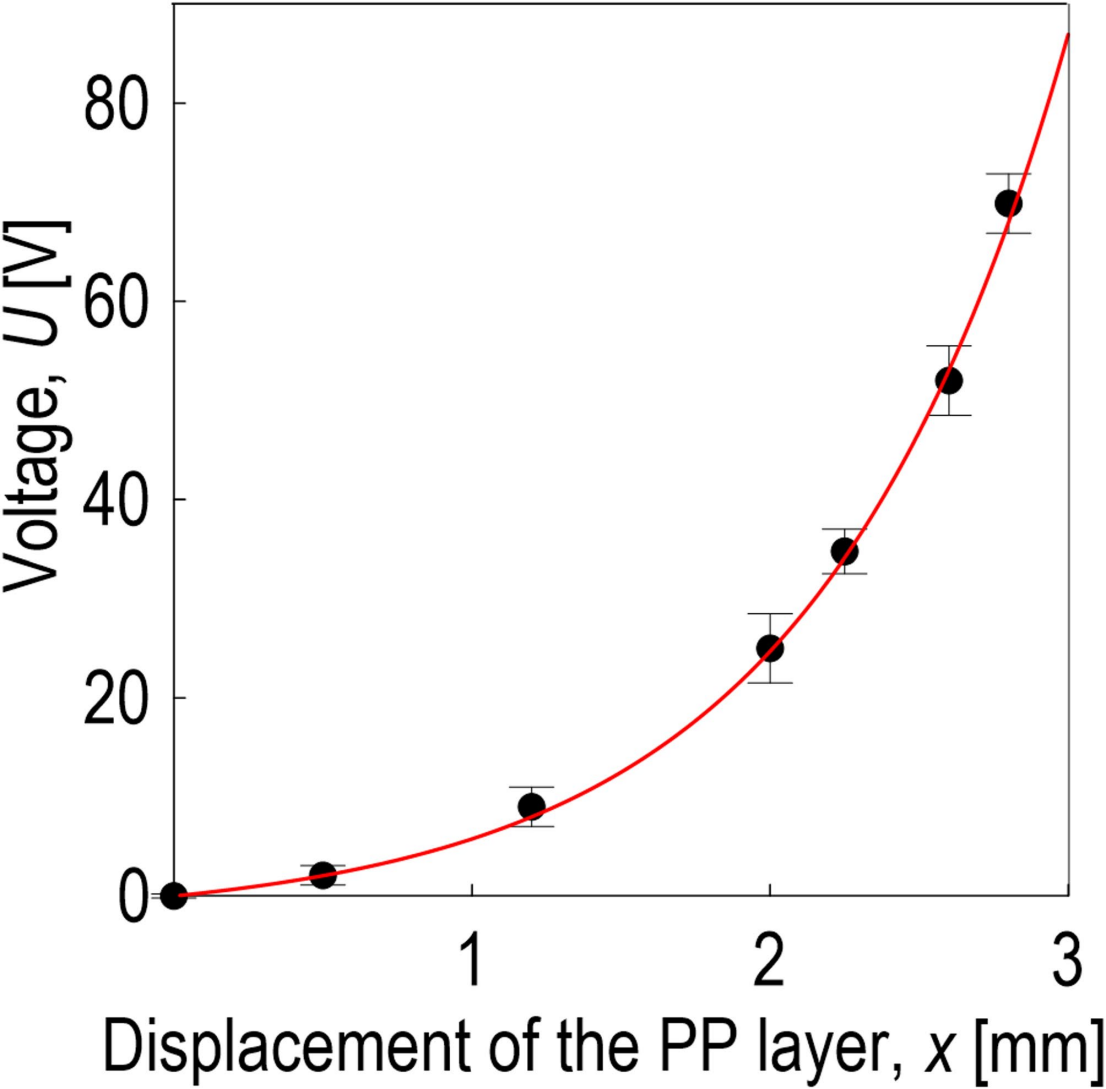
Peak voltage measured at 1 MΩ load as a function of the displacement of the PP layer during non-contact separation of PP/PVDF+BOPET TENG (manual set-up, 1 Hz, 270 kPa, 18 cm^2^).

Measurements performed on the PP/PVDF+BOPET system revealed a strong nonlinear dependence of the measured voltage on the displacement of the PP layer (Fig. 10). The separation distance *x* was varied stepwise in the range from 0.6 to 2.9 mm, while other parameters were kept constant. The corresponding average peak voltages measured (10 times and averaged) at a load of 1 MΩ were 3.6, 9.1, 24.9, 34.8, 52.0, and 69.9 V (Fig. 10). The relation was fitted using the exponential function:

2 $$U = a[{\rm exp}\: (bx)\:-\:1]$$

where *U* is the peak voltage and *x* is the displacement of the PP layer towards the PVDF surface, while *a* and *b* are fitting parameters. The fitting gave *a* = 2.52 and *b* = 1.19 with a correlation coefficient of *R* = 0.989. These results confirm that measurable electrical output can be generated without direct contact between the triboelectric layers and demonstrate the strong sensitivity of the system to changes in the air-gap distance.

### Demonstrations of sensing and energy harvesting

The operation of triboelectric sensor is based on electrostatic induction between surfaces carrying a pre-existing triboelectric charge on the PP and PVDF layers. When a compressive force reduces the distance between the tribopositive PP layer and the tribonegative PVDF, the electric field across gap changes induces a voltage that reflects the displacement of the PP layer.

**Fig. 11:**
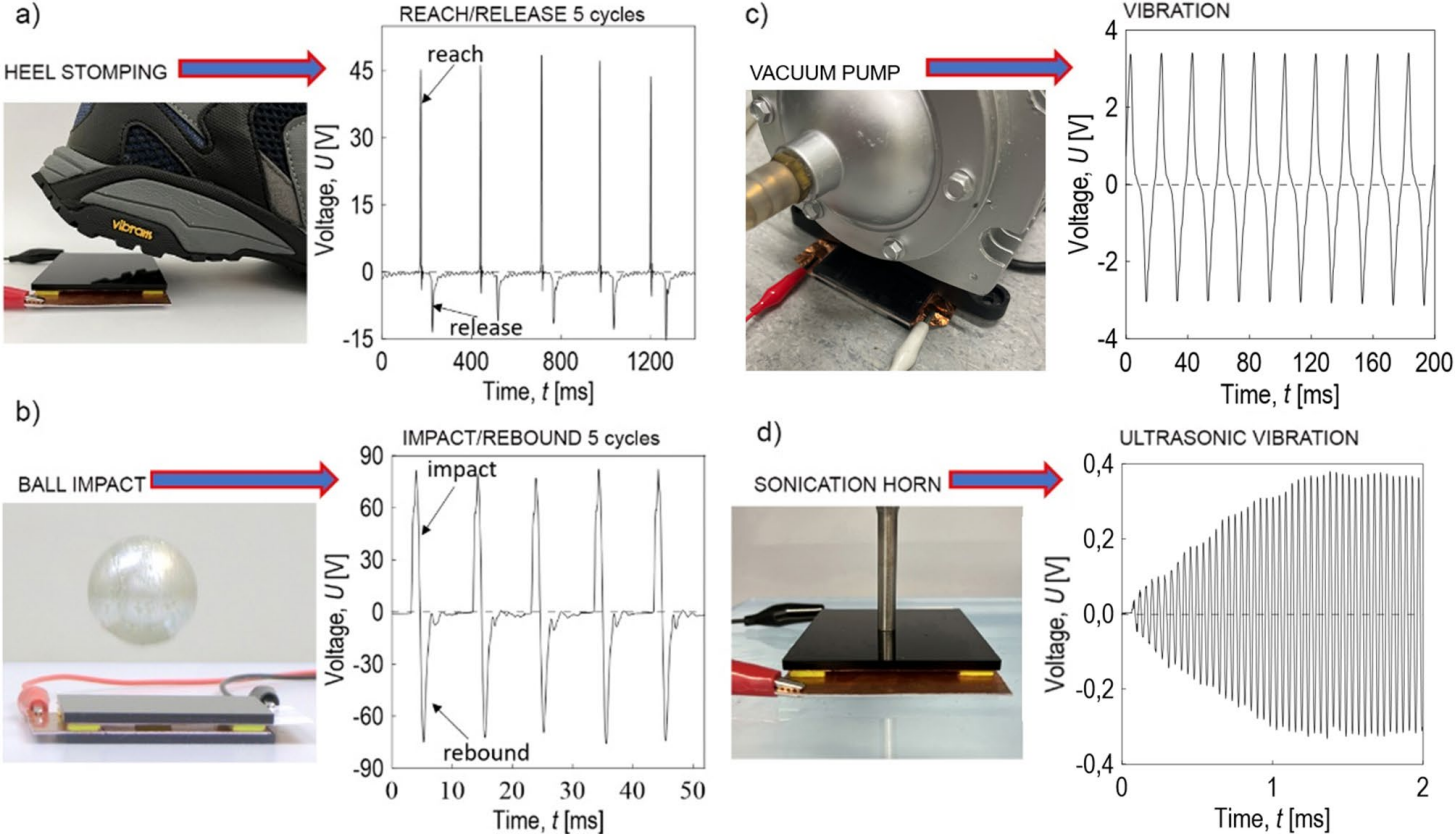
Voltage responses of triboelectrically-induced non-contact PP/PVDF+BOPET sensor to different types of mechanical excitation: (**a**) heel stomping, (**b**) ball impact, (**c**) vibrations generated by a rotary vacuum pump and (**d**) ultrasonic vibration from a sonication horn, where the signal corresponds to the response upon switching on the ultrasonic excitation.

The EVA foam spacers (3 mm) positioned at the corners of the PMMA plates (scheme Fig. 2c), define the nominal distance between the triboelectric layers and control their relative displacement during compression. After the compressive force is released, the spacers act as compliant spring elements and return the plates to their initial position. The sensing performance was evaluated under various mechanical excitations, including cyclic human stepping, impact of a freely falling ball, vibrations generated by a rotary vacuum pump and ultrasonic excitation (Fig. 11). Although the absolute amplitude is small due to micron-scale displacements for the ultrasonic excitation, the ultrasound switch on event is clearly distinguishable from the baseline noise. These experiments demonstrate the ability of the sensor to respond to a wide range of pressure and vibrational stimuli.

**Fig. 12:**
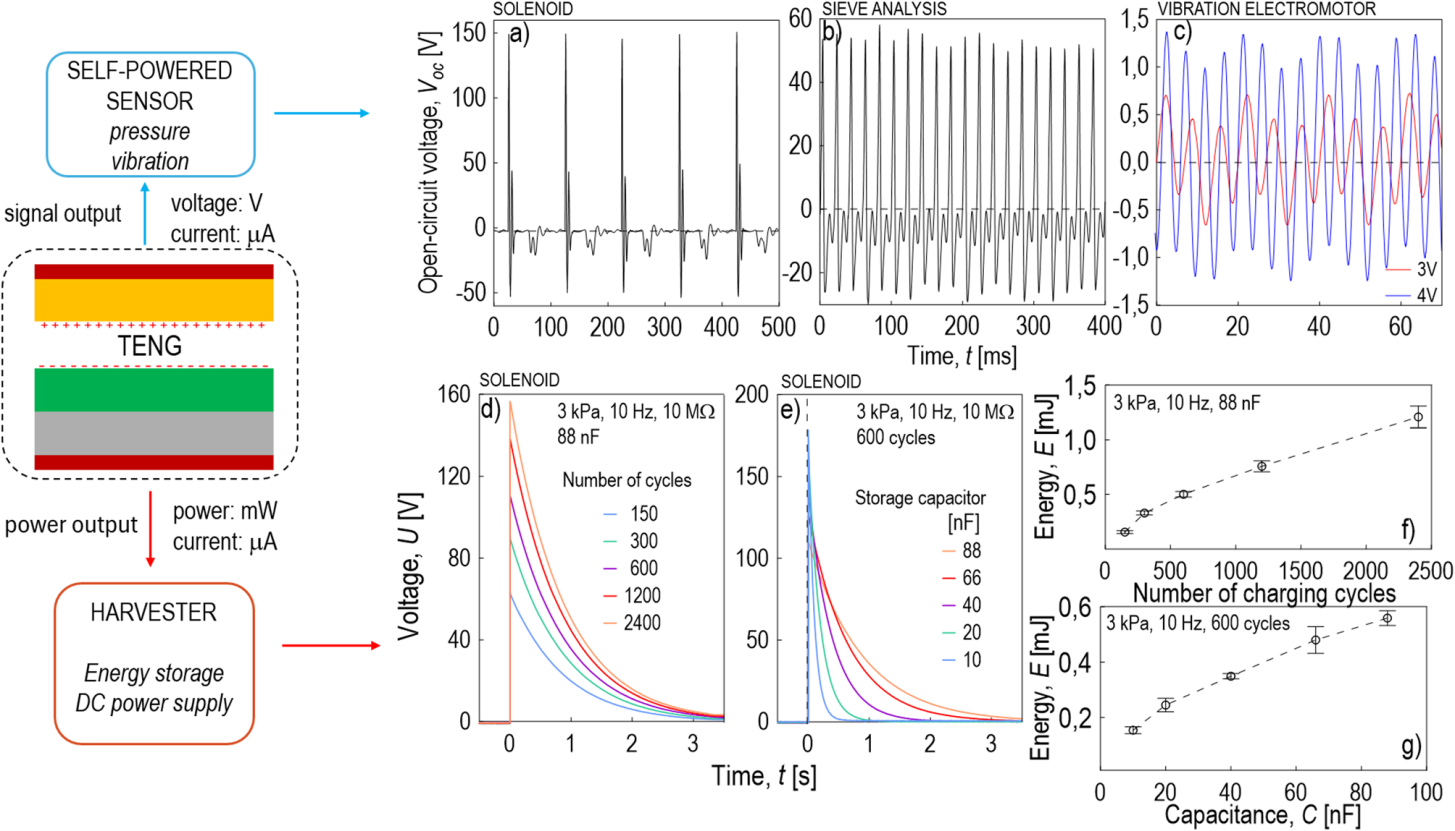
Sensing and energy harvesting performance of the PP/PVDF+LDPE: (**a–c**) representative open-circuit voltage generated under different mechanical excitations, (**d–e**) discharging of storage capacitor driven by rectified triboelectric output: (**d**) voltage as a function of number of cycles at capacitance of 88 nF and (**e**) storage capacitor dependent voltage at 600 cycles, (**f–g**) stored energy as a function of (**f**) number of cycles and (**g**) capacitance.

Figure 12 demonstrates the simultaneous sensing and energy harvesting of PP/PVDF+LDPE device in the non-contact configuration (Fig. 2c). The open-circuit voltage (Fig. 12a–c) retain the characteristic features of triboelectric sensing, reflecting the time profile of mechanical excitation without the need for an external power source. A demonstration of 18 LED lighting is shown in Supplementary Video V1 (solenoid driven, PP/PVDF, 10 Hz, 14.8 kPa), where the rectified output is directly used to drive the LEDs without intermediate energy storage.

The energy harvesting capability is demonstrated by capacitor charging measurements (Fig. 12d–e), which show gradual accumulation of energy in an external storage capacitor. At a fixed capacitance of 88 nF, the stored energy increases with the number of excitation cycles (up to 2400 cycles), confirming the cumulative charge transfer during repeated mechanical actuation. For a fixed number of cycles (600 cycles), the stored energy depends on the capacitance value, reflecting a trade-off between the achievable voltage and storage capacity. The subsequent discharge through a 10 MΩ load confirms that the accumulated energy can be delivered to an external circuit. The stored energy (Fig. 12f–g), estimated from the capacitor voltage and capacitance using *E* = ½*CV*², reaches values in the range of ~ 0.1–1.2 mJ for 88 nF. This indicates that the system can operate simultaneously as a self-powered sensor and an effective energy harvester.

### Interfacial charge retention and activation behavior

**Fig. 13:**
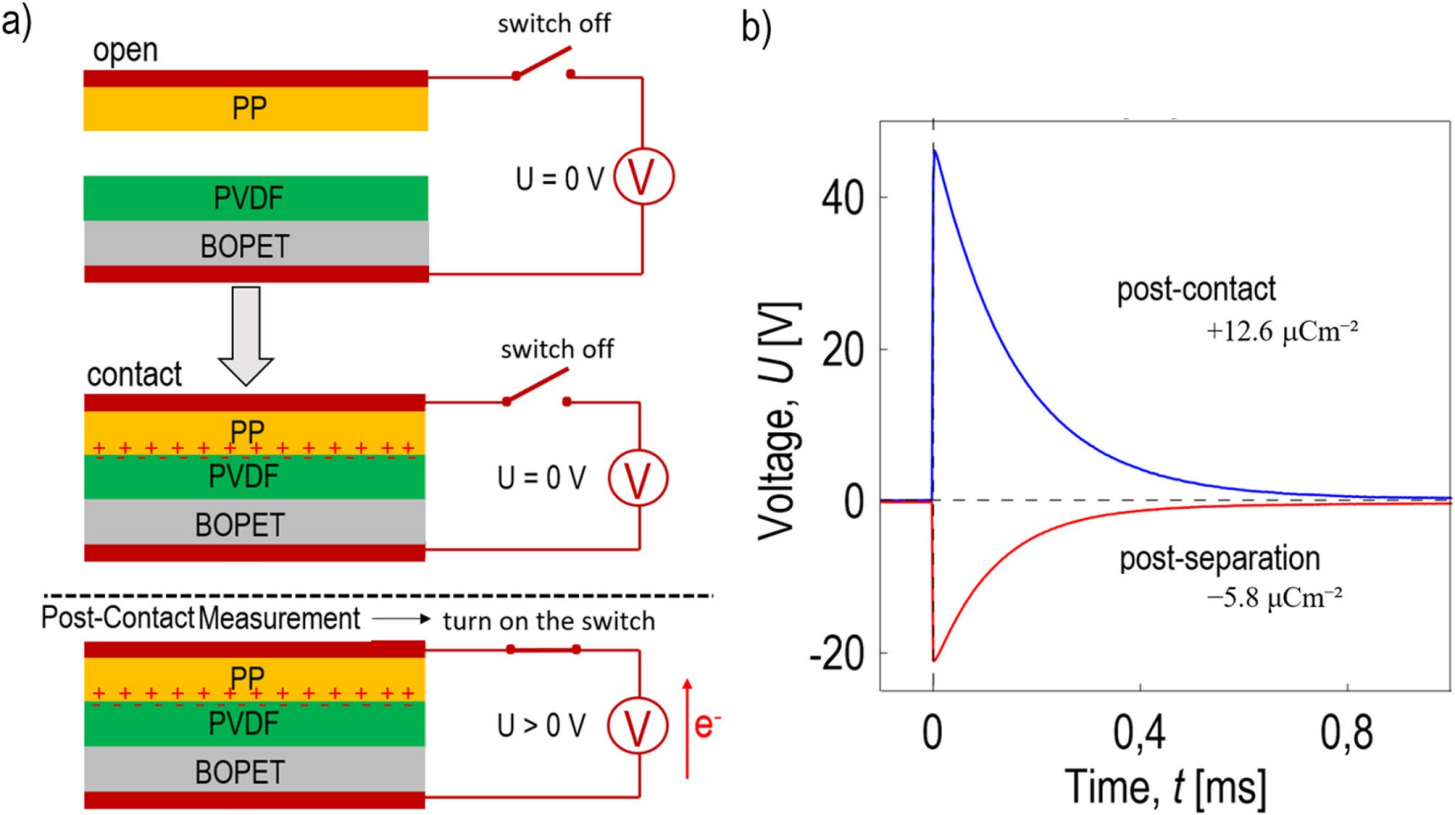
Post-contact and post-separation activation experiments on PP/PVDF+BOPET: (**a**) schematic of measurement procedure showing open-circuit contact and separation, followed by electrical activation after the closure of external circuit, and (**b**) corresponding voltage responses upon circuit closure, demonstrating charge release after contact (positive peak) and after separation (negative peak).

The post-contact and post-separation activation experiments (Fig. 13) provide direct insight into the interfacial charge behavior and show that the triboelectric charge is retained under open-circuit conditions and is released after the circuit is closed.

In the post-contact experiment, the PP and PVDF layers (with a sublayer) were first brought into contact under open-circuit conditions. Prior to contact, the electrodes were short-circuited to remove any residual charge. After approximately 0.5 s in the fully contacted state, the external circuit was closed, triggering charge redistribution and generating a voltage. The resulting signal exhibited an exponential decay characteristic of capacitor discharge with a peak voltage of approximately + 40 V and a corresponding triboelectric charge density of about + 12.6 µC m⁻². This behavior reflects the release of interfacial charge stored during the contact phase.

In the complementary post-separation experiment, the electrodes were short-circuited again before separating the PP and PVDF layers under open-circuit conditions. During separation, the triboelectric charge remained electrically isolated and could not be further redistributed. Once the layers reached the fully separated state, closing the external circuit induced charge flow in the opposite direction, resulting in a voltage transition with a negative peak of approximately − 22 V and a corresponding charge density of about − 5.8 µC m⁻². The subsequent exponential decay confirms discharge of the stored interfacial charge.

These results provide direct experimental evidence of interfacial charge retention and activation. The ability of the system to store charge under open-circuit conditions and release it only upon electrical connection is essential for its operation in a predominantly non-contact regime, where the electrical response is driven by electrostatic induction rather than continuous mechanical contact.

## Discussion

The interfacial charge dynamics of the PP/PVDF system can be interpreted as the result of contact electrification at polymer–polymer interfaces, where charge transfer is governed by differences in electron affinity and surface properties^32,33,36^. After contact, the triboelectric charge accumulates at the PP/PVDF boundary and remains spatially confined due to the absence of a conductive pathway. Under open-circuit conditions, no electrical signal is observed until the external circuit is closed. This charge retention, directly demonstrated by post-contact and post-separation activation experiments (Fig. 13), highlights that the interfacial charge state is defined by the mechanical history of the system and can persist without continuous actuation.

The role of the low-permittivity sublayer is to modify the dielectric environment of the tribonegative side. By reducing the equivalent permittivity of the multilayer structure, the sublayer limits the dielectric screening of the interfacial charge and enhances the electric field reaching the electrode^25–28,31^. Importantly, although the triboelectric charge is generated at the PP/PVDF interface, the resulting electrical output is also strongly influenced by the dielectric properties of the underlying layers, which govern the electric field distribution within the structure. This leads to a higher induced voltage, which is in agreement with both theoretical considerations and previous studies. The observed trend (LDPE > BOPET > PVC) confirms that dielectric permittivity is the dominant material parameter controlling the performance in this system, and that any packaging polymer film with a sufficiently low permittivity can serve as an effective supporting layer without requiring chemical modification or complex processing.

In the non-contact configuration, the electrical response is controlled by the instantaneous separation between the triboelectric layers. The nonlinear dependence of the voltage on the air-gap distance indicates that the electrical response is strongly modulated by the separation between the layers. This enables reliable transduction of mechanical stimuli without the need for continuous physical contact, which is advantageous for sensing applications involving vibration, impact, or acoustic excitation.

The post-contact and post-separation experiments further show that the interfacial charge remains stored and is released through charge redistribution upon electrical connection. The sign of the resulting voltage response reflects the mechanical history of the interface, indicating that the system shows a capacitive-like behavior. This decoupling of mechanical excitation from electrical activation distinguishes the present system from conventional TENG arrangements^37^, where signal generation is typically driven by continuous mechanical cycling.

The behavior of the investigated PP/PVDF triboelectric pair can be also interpreted by combining their triboelectric outputs with the influence of dielectric permittivity on charge induction. Measurements of the triboelectric charge density against a copper reference confirmed that all polymers used in this study are tribonegative with respect to Cu, with PVDF and its network variant exhibiting the highest negative charge densities. PP is less tribonegative, which is consistent with its positive polarity in the PP/PVDF pair.

In the non-contact configuration, the electrical output is governed by the instantaneous separation between the tribopositive (in respect to PVDF^20^ PP layer and the tribonegative PVDF surface The measured voltage–distance dependence exhibits an exponential trend within the tested range, highlighting the strong influence of the air-gap on the induction process. This behavior demonstrates that the PP/PVDF pair can reliably transduce relative plate motion into an electrical signal in a predominantly non-contact configuration. This behavior demonstrates that the PP/PVDF pair can reliably transduce relative plate motion into an electrical signal. This principle is further supported by experiments under various model excitations, including pulsed loading, vibration, and ultrasonic stimulation, where the induced voltage enables both motion detection and low-power energy harvesting.

## Conclusion

A triboelectric sensing system based on the PP/PVDF pair was developed for motion detection and low-power energy harvesting. The system operates predominantly non-contact regime converting variations in the separation distance into electrical signals without requiring permanent mechanical contact between the triboelectric layers.

Introducing low-permittivity polymer sublayers (LDPE, ε = 2.16; BOPET, *ε* = 2.95; PVC, *ε* = 3.14) under the PVDF surface significantly enhances the electrical output by reducing dielectric screening, with LDPE providing the strongest improvement among three sublayers tested: a peak open-circuit voltage of 689 V and a maximum power density of 5.46 mWcm⁻² at the optimal load resistance of ~ 2.2 MΩ under solenoid-driven excitation compared to 376 V and 2.05 mWcm⁻² for the reference PP/PVDF device. The corresponding peak current density for the LDPE configuration reaches 53 µAcm⁻², compared to 31 µAcm⁻² for PP/PVDF. This confirms that dielectric permittivity is a key parameter for tuning the performance of the system.

The sensing capability was demonstrated under various mechanical excitations, including pulsed loading, vibration and ultrasonic stimulation, while post-contact and post-separation measurements revealed that the interfacial charge is retained under open-circuit conditions and is released upon circuit closure.

These findings show that the combination of interfacial charge retention and dielectric field control enables a triboelectric system capable of operating in a non-contact regime while simultaneously providing sensing and energy-harvesting functionality. The presented approach offers a simple and low-cost platform for motion monitoring and self-powered signal generation.

## Supplementary Information

Below is the link to the electronic supplementary material.

Supplementary Material 1

Supplementary Material 2

## Data Availability

DOI: 10.5281/zenodo.18671323.
