# Supplementary figures and images for: Triboelectrically-induced non-contact polypropylene/polyvinylidene fluoride sensor with low permittivity supporting layers affecting its interfacial charge dynamics
Source: Sci Rep. 2026 May 19;16:22783. doi: 10.1038/s41598-026-53473-9 (PMC13385747; doi:10.1038/s41598-026-53473-9)

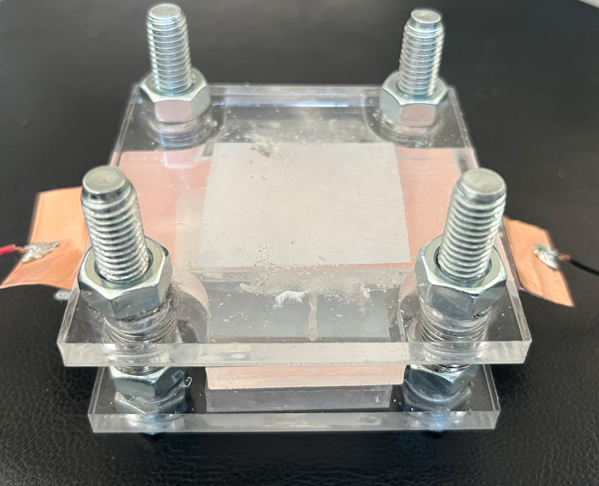

Supplement: Supplementary file 1 — Supplementary Material 1 [file 41598_2026_53473_MOESM1_ESM.jpg]
